# Fiber-to-Chip Three-Dimensional Silicon-on-Insulator Edge Couplers with High Efficiency and Tolerance

**DOI:** 10.3390/mi14081500

**Published:** 2023-07-26

**Authors:** Xiaoyu Li, Shengtao Yu, Chengqun Gui

**Affiliations:** 1 The Institute of Technological Sciences, Wuhan University, Wuhan 430072, China; xiaoyul@whu.edu.cn; 2 School of Power and Mechanical Engineering, Wuhan University, Wuhan 430072, China; yushengtao@whu.edu.cn

**Keywords:** integrated optics, edge couplers, three dimensional (3D), transmission efficiency, misalignment tolerance, fiber-to-chip coupling

## Abstract

The edge coupler is an indispensable optical device for connecting an external fiber and on-chip waveguide. The coupling efficiency of the edge coupler affects the effective integration of optical circuits. In this study, three-dimensional (3D) edge couplers with high efficiency and tolerance are proposed. The high coupling efficiency of the 3D edge couplers is verified by theoretical calculations. Three couplers are fabricated on a thick-silicon platform via 3D grayscale lithography. At the 1550 nm band, the fiber-to-chip experimental data show that the maximum coupling efficiencies of the three edge couplers are 0.70 dB and 1.34 dB, 0.80 dB and 1.60 dB, and 1.00 dB and 1.14 dB for the TE and TM modes, respectively. At the 1550 nm band, misalignment tolerances measurement data reveal 0.8 dB/0.9 dB tolerance of ±5 μm in the horizontal direction, and 1.7 dB/1.0 dB tolerance of ±2 μm in the vertical direction for TE/TM mode. This study provides a new idea for the design of 3D edge couplers and demonstrates significant superiority in research and industrial applications.

## 1. Introduction

In recent years, with the rapidly growing demand for data processing, optical interconnection technology has been developed rapidly [1,2]. The optical signal transmission between the chips needs to be implemented through the optical fiber. Therefore, optical couplers have become basic devices for photonic integration [3,4]. In the preparation of highly integrated optical modules, the direct coupling of the optical fiber and waveguide will lead to a huge mode mismatch loss and reduce the coupling efficiency. Grating couplers and edge couplers can solve these problems [5,6]. Compared with wavelength-sensitive and polarization-sensitive grating couplers, edge couplers can adiabatically adjust the mode spot size, thus effectively coupling with the fibers [7]. Edge coupling has been shown to be an effective way of improving coupling efficiency. Edge couplers exhibit the advantages of intuition, high coupling efficiency, easy integration, and so on [8,9]. Compared with 2D edge couplers, edge couplers with 3D topography can improve alignment tolerances in both lateral and vertical directions [10,11,12]. Additionally, 3D edge couplers can further reduce the mode mismatch loss between the optical fiber and photonic chip, and enlarge the process window of the chip testing and packaging in turn so that the design and manufacture of 3D edge couplers will provide a research basis for large-scale industrialization [13]. Silicon-on-Insulator (SOI) is a mainstream technical material widely used in deep submicron low-voltage, low-power integrated circuits, which holds incomparable advantages over bulk silicon [14]. It is able to realize the dielectric isolation of components in photonic integrated circuits (PICs) and eliminate the parasitic latch effect in bulk silicon complementary metal oxide semiconductor (CMOS) circuits [15]. Photonic integrated devices made of SOI material also present low parasitic capacitance, high integration density, high speed, simple fabrication, and so on [16], making them particularly suitable for low-voltage and low-power circuit design. Optical devices based on SOI can improve the optical switching speed, reduce the power consumption, and realize the characteristics of high-speed operation [17]. Moreover, thick-silicon SOI photonic integrated circuits (PICs) have low optical loss, ultra-high-density integration, low polarization dependence, and the ability to withstand relatively high optical power [18]. The 3D edge couplers based on thick-silicon SOI can realize high-efficiency and high-tolerance coupling [10]. For the study of edge couplers, An et al. experimentally demonstrated two fork-shaped and one dual-trident Subwavelength Grating (SWG)-shaped edge couplers based on a Silicon-on-Insulator platform [19]. The measured results showed that the coupling losses were 1.25 dB/facet, 1.49 dB/facet, and 1.82 dB/facet for fork1, fork2, and dual-trident SWG couplers. Roberto Larrea et al. proposed and optimized a fiber-to-chip spot-size converter (SSC) based on a parabolic inverted taper between two lateral air trenches. The experimental results demonstrated coupling losses as low as 2 dB at the wavelength of 1310 nm [20]. Yisu Yang et al. proposed a multilayer silicon nitride (SiN) PIC at the Communication band (C-band). The butt-coupling loss of 3.7 ± 0.1 dB between an on-chip Distributed FeedBack (DFB) laser and a SiN edge coupler at 1549.48 nm was achieved [21]. Nevertheless, due to the auxiliary thin layers, it is not suitable for large-scale production applications. In addition to the study of the 2D edge couplers proposed above, Qing Fang et al. demonstrated a low-loss and broad-bandwidth 3D functional SiO_2_ taper for silicon photonics [13]. However, its wide application is limited because of its relatively low alignment tolerance [22,23]. Tzu-han Chang et al. reported a 3D tapered waveguide-to-fiber coupler for high-efficiency optical coupling, but whose production process is relatively complex, which does not facilitate mass production [24]. Chun-Wei Liao demonstrated a 3D adiabatic tapered coupler made of polymer; the measured coupler loss was 3 dB. The misalignment tolerance was 3.5 μm and 3 to 5 μm for the lateral and vertical offset based on a 3 dB loss window. Due to its low heat resistance and aging of polymer waveguides, it is not fit for commercial production [25]. W. Zhang presented a silicon and buried oxide 3D edge SSC for low-loss optical mode transition, but its complex fabrication process will increase the production cost [26]. The research of some of the edge couplers mentioned above involves two lithography, two etching, and multiple deposition processes. In addition, their coupling efficiencies and misalignment tolerances are not high. Therefore, based on a thick silicon platform, a 3D edge coupler with novel structure, low production cost, high coupling efficiency, and misalignment tolerance can be proposed.

In this study, three shapes of 3D edge couplers are proposed and fabricated. They are mainly composed of two parts: one is the rectangular ridged waveguide segments at the bottom, and the other is the trapezoid, triangular, and trilateral waveguide segments above them, respectively. The efficient coupling characteristics of the edge couplers for TE and TM modes were verified by 3D finite difference time domain (FDTD) calculations. By using 3D grayscale lithography and dry etching processes, finished edge couplers with a length of 55 μm and cross-sectional sizes of 10 × 4 μm^2^ are obtained. Fiber-to-chip experimental data of the 1550 nm band show that the coupling efficiencies of the 3D edge couplers with trapezoid, triangle, and trilateral at the top are 0.70 dB and 1.34 dB, 0.80 dB and 1.60 dB, and 1.00 dB and 1.14 dB for the TE and TM modes. The 0.9 dB misalignment tolerance in the horizontal direction is ±5 μm for both the TE and TM modes, with 1.7 dB and 1.0 dB penalties of ±2 μm in the vertical direction for both polarizations.

## 2. Design and Simulation

The total insertion loss in the coupling process of the waveguide and optical fiber is an important index for an optical coupling system. When the optical fiber is coupled with the waveguide, the mode field mismatch loss accounts for the largest proportion of the whole coupling loss. Reducing the coupling mismatch loss is the key to reducing the total insertion loss [27,28]. The overlap between the optical fiber and the waveguide is shown in the following equation [27,29].

(1) $$\eta =0.93\left\{\frac{{4\left(\frac{w}{w_{f}}\right)}^{2}}{\left[{\left(\frac{w}{w_{f}}\right)}^{2}+\varepsilon \right]\left[{\left(\frac{w}{w_{f}}\right)}^{2}+\frac{1}{\varepsilon }\right]}\right\}$$

where $w_{f}$ is the half width of the optical fiber mode field, $w_{x}$ is the half width of the waveguide mode field in the *x* direction, and $w_{y}$ is the half width of the waveguide mode field in the *y* direction of the optical waveguide, $w=\sqrt{w_{x}w_{y}}$,$\;\varepsilon =\frac{w_{x}}{w_{y}}$. When the $\frac{w}{w_{f}}=1$, the coupling efficiency is the highest. That is, when the mode fields of the optical fiber and the waveguide can be matched to the maximum extent, a higher coupling efficiency is produced. To realize the efficient coupling of edge couplers, the 3D height gradient design is beneficial for achieving an excellent mode field match with the optical fiber and the adiabatic transmission of the guided mode [30,31]. Therefore, the existing 3D edge couplers can be optimized to improve their coupling efficiency. The tapered length of the edge coupler has a certain influence on the coupling efficiency of the edge couplers [32,33,34]. When the tapered length is overly long, it is not conducive to the integration of large-scale PICs, while when the tapered length is short, it is not conducive to achieving the efficient adiabatic transmission of edge couplers. Hence, the edge coupler can be optimized to a topography and length suitable for coupling with the optical fiber. Previous studies have shown that 3D edge couplers with different shapes can change the energy distributions of the mode spots [35,36]. In this way, by promoting and altering the 3D appearance of the edge couplers, the mode field energy will distribute in the transmission center as far as possible. 

**Figure 1:**
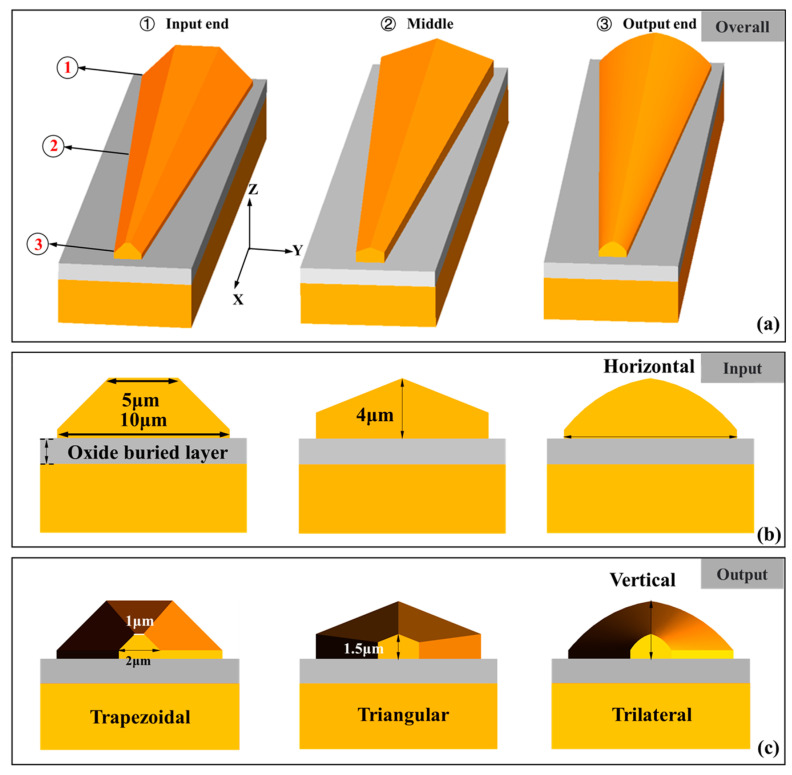
A schematic diagram of the proposed three shapes of 3D edge couplers. (**a**) The overall view of the three 3D edge couplers. (**b**) Side view of the 3D edge couplers from the input end. (**c**) Side view of the three 3D edge couplers from the output end.

**Figure 2:**
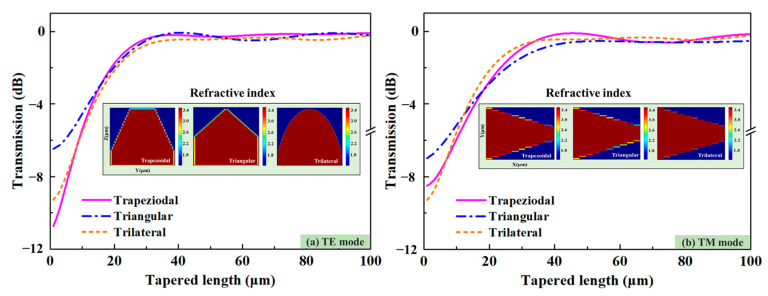
The EME calculation of the coupling efficiency and the refractive index with tapered length within 100 μm. (**a**) Coupling efficiencies and refractive indexes of the 3D edge couplers for TE mode. (**b**) Coupling efficiencies and refractive indexes of the 3D edge couplers for TE mode.

Following the above analysis, the 3D edge couplers with rectangular ridged sections at the bottom, trapezoidal, triangular, and trilateral sections at the top were designed as shown in Figure 1. The thickness of the bottom rectangular ridged section was 1 μm, and the length of the top trapezoidal section was 5 μm. The maximum thickness of the three edge couplers to the embedded silicon dioxide layer was 4 μm. The output size of the three edge couplers was 2 × 1 × 1.5 μm^3^. When the edge coupler was coupled with the optical fiber, the light enters the edge couplers after propagation, it is emitted from the output ends. The design of the rectangular ridges was intended to add aligned and non-aligned coupling-sensitive areas. In order to study the effect of the tapered length on the coupling efficiency, the eigenmode expansion (EME) method was used to simulate and analyze the influence of tapered length and effective refractive index on the coupling efficiency. The calculated results at the 1550 nm band with a sweeping length within 100 μm are shown in Figure 2. It is can be seen that when the tapered length was less than 40/50 μm for the TE/TM mode, the coupling efficiency gradually increased with the aggrandizement of tapered length. The three kinds of edge couplers with different lengths showed different coupling properties for light with different polarized illuminations. The maximum coupling efficiency difference of three edge couplers was lower in TE mode than that in TM mode. When the tapered length was more than 55 μm, the coupling efficiency tended to be stable near their maxima, with their maxima being 0.18 dB and 0.28 dB, 0.31 dB and 0.44 dB, and 0.26 dB and 0.41 dB for TE and TM modes, respectively. This means that when the length of the three edge couplers reaches 55 μm, they will be close to their highest coupling efficiencies. The refractive index distributions of the 3D edge coupler show that the refractive indexes are related to their shapes. To embody the high efficiency and compactness of the 3D edge couplers, the length of edge couplers was set to 55 μm.

## 3. Theoretical Calculation

### 3.1. Coupling Efficiency of the TE and TM Modes

**Figure 3:**
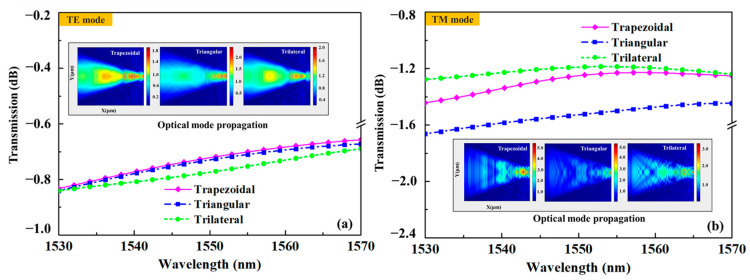
The calculated coupling efficiencies of the 3D edge couplers with wavelengths varying from 1530 nm to 1570 nm. (**a**) Coupling efficiencies and optical mode propagation of the 3D edge couplers for TE mode. (**b**) Coupling efficiencies and optical mode propagation of the 3D edge couplers for TM mode).

**Figure 4:**
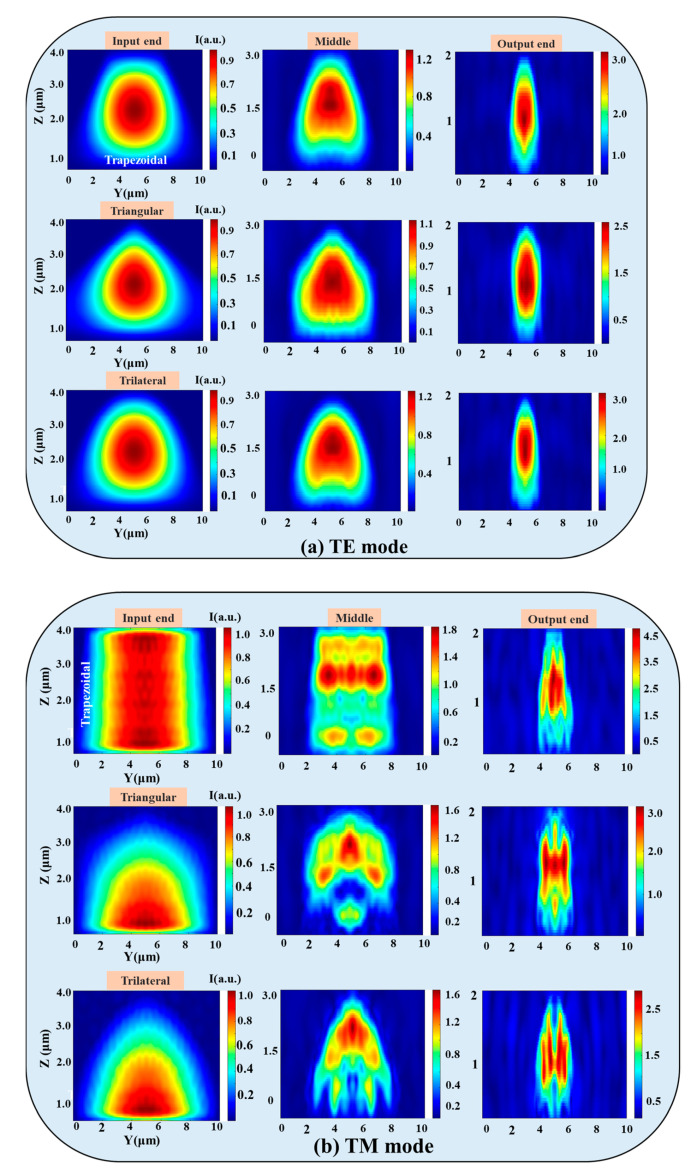
The mode field distributions of the input end, middle, and output end for (**a**) the TE mode and (**b**) the TM mode.

The coupling efficiencies and electric field distributions of the 3D edge couplers in the 1530–1570 nm band were calculated by using 3D FDTD. As shown in Figure 3, the coupling efficiency of the three 3D edge couplers increased with the wavelength. Among them, the coupling efficiency of the trapezoidal edge coupler was the highest, followed by the triangular one, and that of the trilateral one is the lowest for the TE mode, while the coupling efficiency of the trilateral coupler was the highest and the triangular one was the lowest for the TM mode. The coupling efficiencies of the three edge couplers for 1550 nm band are 0.72 dB and 1.25 dB, 0.73 dB and 1.52 dB, and 0.77 dB and 1.19 dB for the TE and TM modes, respectively. The variation ranges of the three 3D edge couplers are within 0.03 dB and 0.04 dB, 0.03 dB and 0.03 dB, and 0.03 dB and 0.02 dB for the TE and TM modes in the simulation band. The results of the FDTD method are basically consistent with those of the EME calculations; that is, the high coupling characteristics of the three 3D edge couplers are verified by theoretical calculations. The electric field distributions of the trapezoidal, triangular, and trilateral edge couplers for TE and TM modes are shown in Figure 4. The mode field distribution during transmission is closely related to the physical shape of the designed edge coupler. The electric field distributions of the three edge couplers show that the shape of the mode spot at the input end, middle, and output end is close to their cross-sectional shape. This phenomenon also illustrates that the 3D edge couplers can realize the high-efficiency propagation of mode fields, especially for the TE mode. The leakage mode was the smallest for the trapezoidal coupler in both the TE and TM modes. Mode leakage was more obvious in the TM mode than in the TE mode, and the mode field intensity of the three edge couplers was stronger for the TM mode than for the TE mode. From the analysis of the transmission efficiencies and the mode field distributions, the calculation indicates that the three 3D edge couplers are equipped with polarization independence.

### 3.2. Calculation of the Misalignment Tolerance

**Figure 5:**
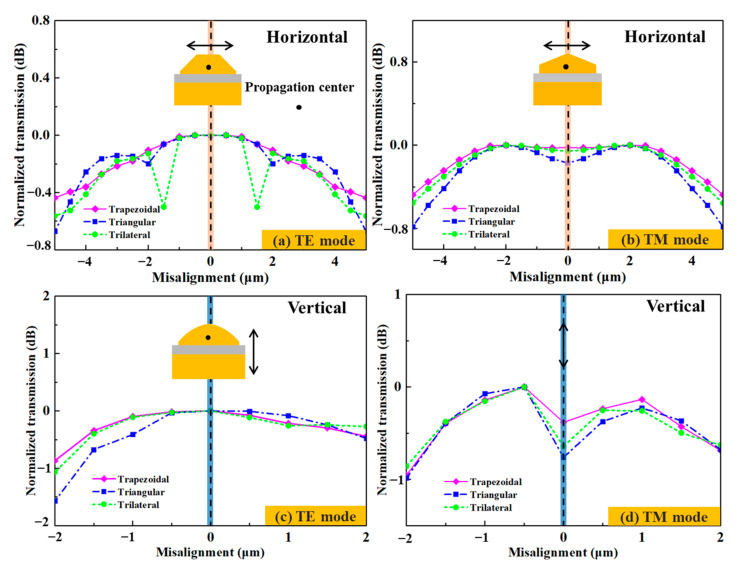
The calculated misalignment tolerances in the horizontal and vertical directions for 1550 nm wavelength.

Misalignment tolerance is an important index for optical chip packaging. The calculated misalignment tolerances in the horizontal and vertical directions for the TE and TM modes of the 1550 nm wavelength can be seen in Figure 5. In the horizontal direction, the coupling efficiency decreases with the distance from the symmetrical position within ±5 μm of both sides of the propagation center. The overall variation tendency of the TM mode is more obvious than that of the TE mode. When the misaligned positions of the TE mode are ±5 μm away from the propagation center, the normalized coupling efficiencies of the trapezoidal/triangular/trilateral edge couplers are within 0.44 dB/0.67 dB/0.56 dB and 0.48 dB/0.78 dB/0.56 dB for the TE and TM modes, respectively. The maximum coupling efficiencies for the trapezoidal, triangular, and trilateral couplers are 0.60 dB/0.69 dB/0.67 dB at the propagation center for the TE mode, while the same values for the TM mode are 1.17 dB/1.37 dB/1.41 dB ±2 μm away from the propagation center. In the vertical direction, the trapezoidal, triangular, and trilateral edge couplers achieve their maximum coupling efficiencies of 0.66 dB, 0.69 dB, and 0.67 dB at the propagation center for TE mode, while 0.81 dB, 0.78 dB, and 0.79 dB are obtained at 0.5 μm below the propagation center for TM mode. The 1dB, 1.6 dB, and 1.1 dB misalignment tolerances of the trapezoidal, triangular, and trilateral edge couplers in the vertical direction are ±2 μm for both the TE and TM polarizations. The optimal coupling positions for the TE and TM modes are different, which also indicate that the three edge couplers have different coupling performances for both polarizations. Among the three edge couplers, the trapezoidal one shows the best coupling performance, followed by the trilateral one. This is because the shape of the 3D edge couplers can affect the mode-filed energy distribution during the optical propagation. The more favorable a shape is for the mode field energy to be closer to the propagation center, the less leakage mode is generated. The more favorable transmission of the guide mode, and the higher coupling efficiency of the edge couplers will be. Therefore, the trapezoidal coupler is the optimal shape, followed by the trilateral and triangular ones, successively. Following the above analysis, more 3D edge couplers with much higher coupling efficiency can be proposed.

## 4. Fabrication and Measurement 

### 4.1. Device Fabrication

**Figure 6:**
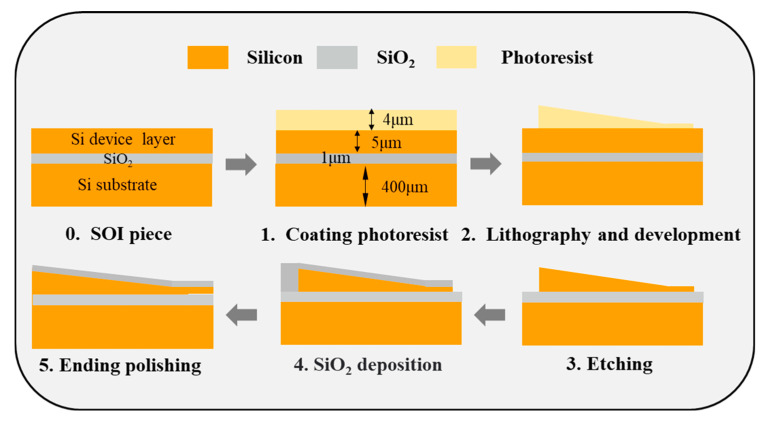
Fabrication process of the 3D edge coupler.

**Figure 7:**
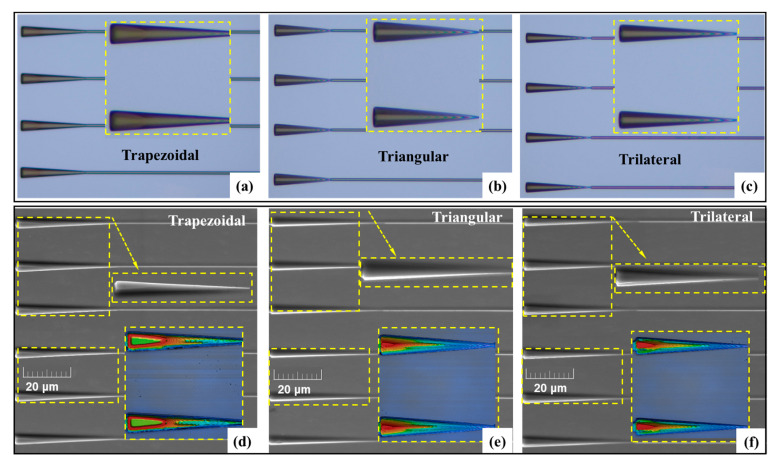
Optical microscope images of (**a**) trapezoidal edge coupler, (**b**) triangular edge coupler, and (**c**) trilateral edge coupler after development; SEM and confocal microscopy images of the (**d**) trapezoidal, (**e**) triangular, and (**f**) trilateral edge coupler after etching.

The 3D edge couplers were fabricated on a 5 μm thick top silicon device layer of SOI with a 1 μm thick SiO_2_ buried layer and a 400 μm thick silicon handle layer. The main fabrication steps are depicted in Figure 6. Firstly, the positive photoresist was coated on the thick-silicon layer of SOI. The layout of 3D edge couplers was imported into the lithography machine in advance to realize the grayscale exposure. After lithography, the sample was developed with an appropriate concentration of developer. The optical microscope images of the 3D edge couplers after development are shown in Figure 7a–c. The Inductively Coupled Plasma-Reactive Ion Etching (ICP-RIE) technique was applied to etch the developed samples. The Scanning Electron Microscope (SEM) and confocal microscope images of the etched 3D edge couplers are shown in Figure 7d–f. Then, the SiO_2_ cladding with a thickness of 800 nm was deposited on the top silicon layer by using Plasma-Enhanced Chemical Vapor Deposition (PECVD) technology. The input end was successively polished with the Chemical Mechanical Polishing (CMP) process. At this time, the 3D edge coupler was completed, and the fiber-to-chip coupling testing was conducted after the sample was sliced.

### 4.2. Validation and Analysis

**Figure 8:**
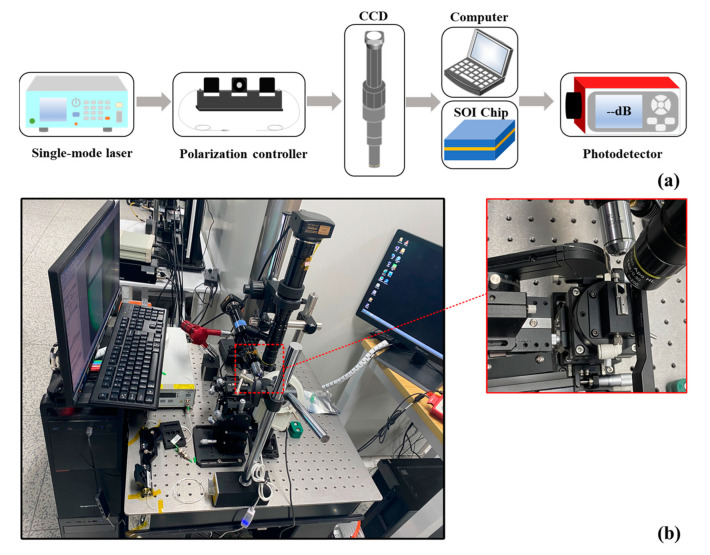
(**a**) The schematic diagram of the test equipment; (**b**) the fiber-to-chip measurement setup on the optical platform.

**Figure 9:**
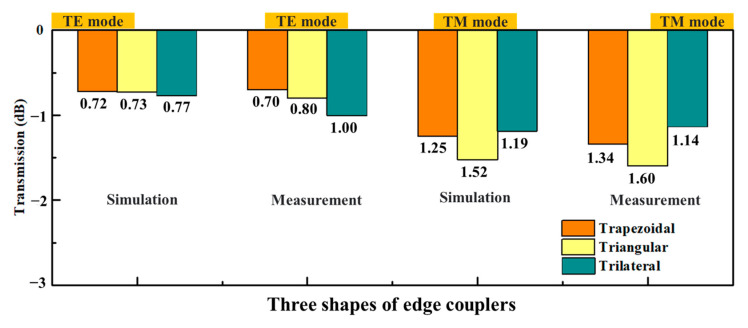
A comparison of the simulated and measured results for 1550 nm wavelength.

The fiber-to-chip coupling test was used to measure and validate the coupling performance of the 3D edge couplers. In order to ensure the alignment accuracy, all the test devices are placed on an optical platform equipped with air flotation to maintain the stability of the equipment. The fiber-to-chip testing setup is shown in Figure 8. By adjusting the position of the translation table, the optical fiber and the SOI chip will be in the best aligned state. After the light source was emitted from the single-mode laser and controlled by the polarization controller, it was coupled to the edge couplers through the lensed fiber (with a diameter of 5 μm), which was in contact with the matching liquid (the refractive index was 1.45). After the light was shed from the output end of 3D edge couplers and received by the photodetector, the measured coupling efficiency could be obtained. The comparison between the simulated calculations and measured results of the three edge couplers for 1550 nm band is shown in Figure 9. The measured data of the trapezoidal, triangular and trilateral couplers were 0.70 dB and 1.34 dB, 0.80 dB and 1.60 dB, and 1.00 dB and 1.14 dB for the TE and TM modes. The measured results of the 1550 nm band were in good agreement with the theoretical calculations, which verified the efficient coupling characteristics of the three edge couplers.

**Figure 10:**
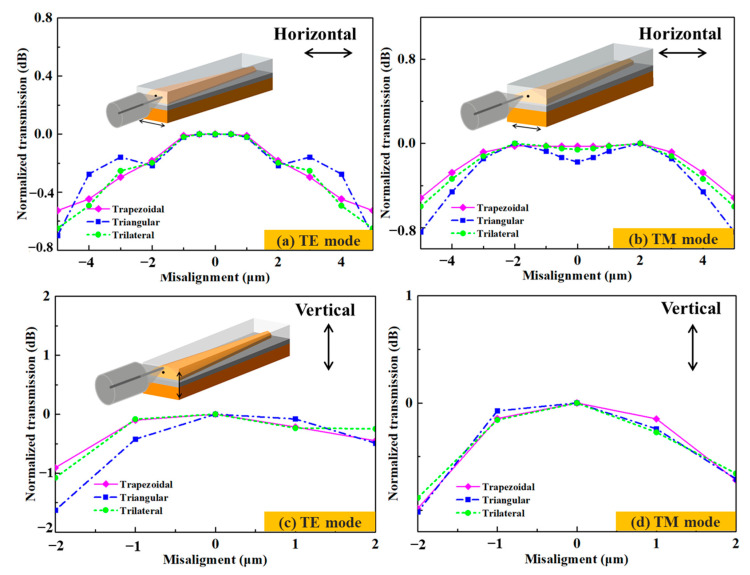
The measured misalignment tolerances in the horizontal and vertical directions for 1550 nm wavelength.

The misalignment tolerance data of the 3D edge couplers are shown in Figure 10. When coupled in the horizontal direction, the measured coupling efficiencies of the trapezoidal, triangular and trilateral edge couplers were within 1.2 dB/1.8 dB, 1.5 dB/2.4 dB, and 1.4 dB/2.2 dB for the TE/TM mode ±5 μm farther from the propagation center. When coupled in the vertical direction, the measured coupling efficiencies of the three edge couplers were within 1.7 dB/2.0 dB, 2.5 dB/2.0 dB, and 2.0 dB/1.8 dB for the TE/TM mode ±2 μm away from the propagation center. The misalignment tolerances of the three edge couplers were 0.8 dB and 0.9 dB penalty ±5 μm in the horizontal direction for both the TE and TM modes. The 1.7 dB and 1.0 dB misalignment tolerances in the vertical direction were ±2 μm in the vertical direction for both polarizations. The optimal coupling positions in the horizontal and vertical directions were also consistent with the simulated calculations. 

## 5. Conclusions

In conclusion, three 3D edge couplers were proposed and fabricated on the thick-silicon layer of SOI. The efficient coupling characteristics between the lensed fiber and edge couplers were verified by EME and FDTD calculations. The 3D edge couplers were fabricated using 3D grayscale lithography and ICP-RIE etching processes. The fiber-to-chip experimental data for the 1550 nm band indicate that the coupling efficiencies of trapezoidal, triangular, and trilateral edge couplers are 0.70 dB/1.34 dB, 0.80 dB/1.60 dB, and 1.00 dB/1.14 dB for TE/TM modes. The 0.8 dB and 0.9 dB misalignment tolerances in the horizontal direction are ±5 μm from the propagation center for the TE and TM modes; the 1.7 dB and 1.0 dB penalties in the vertical direction are ±2 μm away from the propagation center for both polarizations. The simplified fabrication and compact 3D edge couplers are beneficial for the integration of macro-chips. The high misalignment tolerances of the 3D edge couplers can bring convenience for the packaging of photonic chips. Through further optimization, standard single-mode 3D edge couplers with higher coupling efficiency can be manufactured and implemented in large-scale PICs.

## Data Availability

Data will be made available on request from the corresponding author.
